# The Insult of Involuntary Adoption and the Moral Seriousness of MOTHERHOOD

**DOI:** 10.1002/hast.4957

**Published:** 2025-03-05

**Authors:** Katie Watson

**Keywords:** abortion, motherhood, coercion, bioethics, antiabortion legislation

## Abstract

*Adoption is often framed as an alternative to abortion. However, many women feel that pregnancy and birth made them a mother and that this new identity is not erased by the fact they are not raising that child. This article argues that involuntary adoption occurs when legislative coercion deprives a pregnant person of a realistic abortion option, forcing them into this position of being a parent who is not parenting. The article also argues that the* moral seriousness of motherhood *begins at conception, not because embryos are the moral equivalent of babies, but because that's when the pregnant person becomes a “potential mother” who must make a new set of decisions in response to her status. The reversal of* Roe v. Wade *has made narrative insight into the dynamics of pre‐*Roe *adoption critical. Therefore, this article offers the stories of a 1969 birth mother's journey from being a teenager who relinquished her birth daughter to becoming a physician working in abortion care and of a 1965 adoptee's effort to reach out to her birthparents at age fifty‐four as texts for ethical analysis*.

## Article

When the Supreme Court reversed *Roe v. Wade* in 2022, the majority wrote that state legislators and voters can now consider arguments in favor of abortion bans. Among these arguments is the claim that the option of adoption makes abortion unnecessary. The majority stated that pregnant women have “little reason to fear that the baby will not find a suitable home,” and in oral arguments, Justice Amy Coney Barrett suggested that safe‐haven laws—which allow people to anonymously drop off newborns without a formal adoption process—“take care of” the problem of pregnant people wanting to avoid forced parenthood.

It is easy to picture our post‐*Roe* system of voluntary adoption when discussing abortion bans. That's a mistake, because the choice to place a newborn for adoption is voluntary only when abortion is a realistic option. Before *Roe*, the United States had what could be called “involuntary adoption,” and the “adoption‐is‐the‐answer” argument ignores the often painful experiences of the 1.5 million women who placed their babies for nonfamily adoption between 1945 and 1973.^1^ Every woman of reproductive age today was born after 1973, yet it has suddenly become essential for the post‐*Dobbs* generation to understand this little‐known aspect of the pre‐*Roe* experience.

## Case 1: A Pre‐*Roe* Birth Mother

Maureen Paul is a best‐case scenario for those who claim that adoption makes abortion bans ethical. In 1969, she was a student at Michigan State University who couldn't get the illegal abortion she wanted, so, at age nineteen, she dropped out of college, carried her unwanted pregnancy to term, and relinquished her baby for adoption. Later, she joyfully chose motherhood on her own terms, finished college and medical school, and became a professor at Harvard University. When her birth daughter, Deanna, was a twenty‐year‐old student at MSU herself, they reunited on campus. They've been close ever since, and they love each other very much. 

Their story sounds like it was plucked from an anti‐choice campaign video: a happy family made possible only because the law kept Maureen from getting the abortion she wanted. So why is Maureen's teenage experience of involuntary adoption one of the reasons she has chosen to perform roughly 100,000 abortions in her career as an obstetrician‐gynecologist? And why is her birth daughter, who would not exist if abortion had been legal in 1969, a staunchly pro‐choice woman who supported the amendment Michigan voters added to their state constitution in 2022 to protect abortion rights? To answer these questions, I met with Maureen, Deanna, and Dillon (Maureen's daughter by choice) when they gathered for a weekend visit at Deanna's house in a woodsy suburb of Detroit, because a new group of U.S. women is at risk for living the twenty‐first‐century version of their experience.

Maureen was a college sophomore studying to be an English teacher when she got pregnant the first or second time she had sex. She tried to arrange an illegal abortion, but the family friend she asked for the three hundred dollars she needed told her parents, and her mother flew to Michigan from Massachusetts to drag her home. Maureen remembers sitting at their kitchen table while her dad, a punishing man that the kids called “The Captain” behind his back, was apoplectic and called her a whore. Her father took her to a local hospital to ask for a legal, “therapeutic” abortion, thinking her age might sway the committee to approve it, but the committee refused. Maureen was too terrified of self‐abortion to try it—that seemed suicidal to her. Her father said she would have to hide her pregnancy at a home for unwed mothers or marry her boyfriend, Jim. Jim came to Massachusetts to marry Maureen, but he refused her father's demand that he first cut his long hair, and that led to a fight that ended with the couple leaving for Canada. But in 1969, minors were subject to parental control until age twenty‐one, and a telegram from her sister saying, “Get married by Tuesday or Dad gets police,” prompted a courthouse wedding.

The couple went back to Michigan, Jim dropped out of college and got a job at the railroad, and they arranged an adoption because, Maureen explained, there was “no way that we could do this.” Her parents had stopped speaking to her, Jim was pushing adoption in a way that convinced Maureen he wouldn't help parent, and, she said, “I didn't want my life at age nineteen to be about becoming a mother and not finishing school.”

## The Moral Seriousness of Motherhood

Abortion is often framed as a serious moral choice. Deciding to bring a new person into the world is also a serious moral choice, yet we rarely talk about it that way.

The moral seriousness of bringing a new person into the world is ignored by antiabortion adoption arguments. But it's odd that it's also ignored by bioethicists. Our field has argued that creating babies using assisted reproductive technology (ART) raises ethical issues because ART causes a baby to be born who otherwise would not be here and this process involves doctors, procedures, drugs, and laboratories. Yet the way our field treats pregnancy created through intercourse as a fait accompli curiously ignores the doctors, procedures, drugs, and laboratories involved in the medical revolution in contraception and pregnancy termination. Every reader of this journal can probably tell the “new technology” story that also applies to end‐of‐life care—twentieth‐century advances in medicine redefined cultural ideas and practices, and laws changed to accommodate the use of those advances, from determining that withdrawal of life‐sustaining interventions isn't murder or suicide to a brain‐death standard that now allows someone with a beating heart to be declared dead. On a roughly parallel timeline, new medical advances creating reliable, physician‐prescribed contraception and incredibly safe, effective abortion procedures also redefined cultural ideas and practices at the beginning of life, and laws also changed to accommodate their use. Medical advances have made pregnancy and childbearing after intercourse a decision rather than the default for any woman^2^ who believes that contraception or abortion is morally appropriate for her, and who has access to it. Yet this “new technology” narrative is curiously absent from mainstream bioethics analysis and abortion conversation.

Correcting the mistaken view that abortion is an ethical issue but childbearing is not begins with recognizing what I call the “moral seriousness of motherhood,”^3^ which includes a profound duty to do right by your child. Naming this is simply giving voice to something many people already consider. In the past, if you couldn't—or didn't want to—“do right” by your child, placing them for adoption was the decent thing to do. Now that abortion is safe and effective, it is another route to avoid becoming a parent who doesn't have what they need to meet their child's material or emotional needs. In 1987, theologian Virginia Ramey Mollenkott used the term “covenant of caring” to describe something similar: “Pregnant women are faced with deciding whether they can maintain a covenant of caring with the fetus they have conceived. Since a newborn infant needs not only physical care but also psychological attention and tender affect, and since the covenant with a child is life‐long, only the woman involved is capable of determining the probability of living up to that covenant—or, should she give the baby up to adoption, whether she can endure the life‐long anxiety about her child's welfare.”^4^

Recognizing that medical advances allow fertile people and bioethicists to more actively consider the moral questions of whether and when we should have children has nothing to do with governmental control of reproduction. In states that do not ban abortion, decisions to have children are in the same realm of private decision‐making as decisions not to have children. In states that ban abortion, framing childbearing as a private moral choice highlights the immorality of forced childbearing.

From the outside, it looks as though Maureen had a “choice”: she could have raised her baby with her teenage husband, but instead, she chose adoption. “No, I chose abortion,” Maureen insists. To Maureen, every wanted abortion denied is a forced pregnancy and a violation of human rights. “To force a person to carry a pregnancy and then adopt out rather than give up her aspirations in her life and her career, that's not only a violation of her bodily integrity; it's coercion.”

Maureen says the experience of carrying a pregnancy against her will “was as if all the fairy tales of my childhood with their Prince Charmings and happy endings had been rewritten to mock me.” No longer a college student, she spent the last five months of her pregnancy “playing housewife,” cooking, cleaning, and crying. After work, Jim and their friends “would sit in the living room and laugh and talk … and I'm out here in my parallel universe peering in, feeling so alone” as she watched her body change and grow.

The weeks following the birth of the baby she named Celeste were “torture” for Maureen because the adoption agency told her that she had several weeks to change her mind after the birth. “It was very hard to be in proximity and keep saying, ‘Don't go, don't go, don't go, don't go,’ just knowing in my rational mind that I couldn't change my mind, I didn't want to change my mind.”

Shortly after the adoption, Maureen and Jim divorced, and she turned to drugs. “I was completely untethered,” Maureen said. “I think I was fighting depression” with lots of speed and a potpourri of other drugs. “I was self‐medicating, because I had no idea what my life was about.” She thought the drugs might kill her. “It just terrified me.”

## Deepening Our Understanding of Coercion

Maureen's story is consistent with that of most of the women Ann Fessler interviewed for her revelatory book of oral histories, *The Girls Who Went Away: The Hidden History of Women Who Surrendered Children for Adoption in the Decades before* Roe v. Wade.^5^ For thousands of young women who had to involuntarily surrender their babies before *Roe*, adoption “was not a question of choice but of doing what society demanded—a demand that society has never fully acknowledged.”^6^ Unmarried pregnant women were coerced into adoption by family, society, and economics—and then told to move on and forget what had happened. But none of the mothers Fessler interviewed “was able to forget. Rather they describe the surrender of their child as the most significant and defining event of their lives.”^7^

The word “coercion” needs critical attention. In bioethics, the focus of “coercion” is individual action—a manipulative clinician or an abusive family member leads a patient to make a medical decision they wouldn't otherwise choose. Ethicist Bernard Lo, in his widely used textbook, describes coercion as a form of overpowering that “involves threats that are intended to control patients’ behavior and that patients find irresistible,” and he states that this unethical use of coercion or manipulation negates autonomy and vitiates informed consent.^8^ Other barriers can limit a person's medical options, but when they don't involve human agency, we don't classify them as “coercive.” If a blizzard kept a pregnant woman from going to the hospital, we wouldn't say she “chose” an unattended homebirth, but we also wouldn't call the blizzard “coercive.”

Jill Fisher offers the helpful term “structural coercion” to move the analysis of the voluntariness of research participants beyond the researcher‐subject dyad so that we can consider the social and economic contexts that motivate human‐subject enrollment.^9^ Naming how poverty or inadequate access to health care can limit research subjects’ lived experience of “choice” is an important step forward. However, because the term “structural” doesn't assign agency, circumstances like lack of health insurance are naturalized in a way that puts them in the same category as the blizzard. I will refer to legal restrictions on pregnant people's choices as “legislative coercion” to highlight that they indisputably involve human agency and to argue that this policy‐driven coercion deserves more attention from bioethicists. Legislative coercion is an example of collective agency rather than individual agency. These legislative choices are an expression of the agency of the majority of your neighbors—or, in the case of legislative capture, the agency of a minority of your neighbors with disproportionate legislative influence.

The drop in adoption rates after *Roe* suggests the legislative coercion that was exerted through criminal law. Abortion became legally available in 1970 in New York, Washington, Alaska, and Hawaii and, in 1973, nationwide. Between 1970 and 1975, the number of white women placing their babies for adoptions by nonrelatives dropped 63 percent, and for nonwhite women,^10^ it dropped 10 percent.^11^ The Hyde Amendment, which became law in 1977 and prohibits federal funding for abortion in almost all cases, including dollars spent in the public insurance program Medicaid, provides an example of legislative coercion through civil law. Medicaid is a federal‐state partnership, and most state legislatures have chosen to copy the federal policy of prohibiting Medicaid coverage for abortion. A pre‐*Dobbs* study that surveyed Louisiana women in prenatal‐care clinics who were insured by Medicaid concluded that the state and federal policy choice of paying for childbirth but not abortion led three thousand impoverished women per year in Louisiana to deliver babies they would otherwise not have carried to term.^12^ In this example, the Louisiana legislature is playing the role of the abusive husband or manipulative clinician, and its deliberate choice of economic coercion is forcing these patients to remain pregnant when they do not want to be.^13^

Today, the Louisiana legislature is attempting to do the same thing for women in all economic groups by banning abortion altogether. When scholars and advocates describe abortion bans as policies of “forced childbirth,” they are naming the coercive effect of the criminal law. Similarly, my introduction of the term “involuntary adoption” is meant to identify an additional coercive effect of abortion bans. Janet Mason Ellerby, who was a teenage birth mother before *Roe* and went on to become an English professor, describes her experience: “Like so many middle‐class girls in the ‘baby scoop’ era, I felt powerless to determine my fate and so ‘agreed’ to all the arcane machinations that kept my pregnancy a secret. I was not pilloried like Hester Prynne, but I was coerced into believing I was unfit for motherhood, that I had no choice but to surrender the infant whom I loved instantly with a ferocity that still surprises.”^14^

Sociologist Gretchen Sisson has picked up Fessler's baton, interviewing seventy‐two women who relinquished infants for domestic private adoption between 2000 and 2020,^15^ and she and many of the women she interviewed also use the word “coercion” when describing pressure to relinquish children. Although abortion was a constitutional right in this period, many of her subjects describe either poverty or the religious families they lived in, and sometimes adoption agency policies, as “coercive” factors in their inability to act on their desire to parent (the wish of the majority) or obtain an abortion. Poverty is likely to play a role in the surprising fact that a majority of Sisson's subjects were already parenting other children at the time of their relinquishment. Similarly, 59 percent of U.S. abortion patients are already mothers, and 73 percent cite “can't afford a/another baby” as one of their reasons for terminating a pregnancy.^16^

During Maureen's career as an ob‐gyn, the reproductive justice movement gave her new insights on the language she uses with her patients. “I don't use the word ‘choice.’ I always use the word ‘decisions.’ I think it takes a lot to have a real choice.”

## When Does the Moral Seriousness of Motherhood Begin?

When Maureen was at her lowest, a friend she had made in college called. She was in a Seattle women's health collective that was starting a self‐help women's clinic, and she encouraged Maureen to join them. “That phone call saved my life,” Maureen said.

Most of the women who tell their stories in *The Girls Who Went Away* were shamed into keeping their secret. In contrast, Maureen thinks that the women's movement of the 1970s is the reason her life turned out much better than those of most of Fessler's interviewees. The movement gave her an early opportunity to discuss and reframe the oppression she experienced. Maureen moved to Seattle and joined a women's consciousness‐raising group, “which was so, so transformative,” Maureen said, “it brought things into focus for me.” She joined her friend's health collective, and they hosted weekly women's self‐help sessions on reproductive health at the Open Door Clinic. A young ob‐gyn who worked at this free clinic taught them to do pelvic exams, and “we'd have these Friday night period parties, and we'd aspirate each other's periods so we could learn to use manual vacuum aspirations,” Maureen recalled, laughing. They also served as what are now called “standardized patients” to teach University of Washington medical students how to do pelvic exams “because we were so committed to teach them in a different way how to respect women and to have women‐centered care. So, it was amazing. And that's how I got interested in women's medicine.”

In 1972, Maureen and a male friend went to look for her ex‐husband, Jim, who had disappeared while hitchhiking across the country and would later be declared legally dead, and Maureen became pregnant after she and the friend slept together. “I remember kind of intentionally not using birth control,” and because living in a women's collective meant she wouldn't have to parent alone, she thought, “I'm going to make up for this experience” of her first pregnancy and the involuntary adoption. She delivered Dillon in a home birth with friends from the free clinic, and because the collective helped Maureen raise Dillon, she was able to finish college in Seattle while she was a single mother on welfare.^17^ Later, the collective helped her pay medical school application fees.

One evening in 1990, when Maureen was an ob‐gyn in Boston and Dillon was eighteen, the phone rang. It was Deanna, who, after some confusion, blurted out, “I'm your daughter.” Maureen had always updated the adoption agency on her address because “a part of me hoped she would contact me.” Maureen had told Dillon about her first daughter, and Dillon vividly remembers how excited her mother was as Dillon watched her answer Deanna's call.

Earlier, I argued that bioethicists should be more attentive to the moral seriousness of motherhood. But when does that begin? Option 1 is when a pregnant woman becomes a “mother,” which the typical dictionary defines as “a woman in relation to a child or children to whom she has given birth.”^18^

Women who have abortions do not want to have a child they created but are not raising in the world. Abortion avoids this situation. Adoption creates it. Adoption makes sense as a “solution” to abortion only if you think embryos have the same moral status as babies. Locating the beginning of the moral seriousness of motherhood at birth reflects the dueling positions in the debate over embryonic and fetal personhood. Women who seek an abortion do not think their embryo or fetus has the same moral status as a baby. Therefore, one might assume that they are voting with their feet to say that the moral seriousness of motherhood begins at birth—or perhaps at viability or some other later point—but not at conception.

Option 2 is that the moral seriousness of motherhood begins at conception. The antiabortion version of this claim is the familiar one: people who think abortion is unethical often argue that an embryo has the same moral status as you and me (often called the “substantial identity” view) and that pregnant people therefore assume the maternal obligation to not kill their children at conception. They would argue that adoption, not abortion, honors the “covenant of caring” by allowing life to continue and handing the parenting to someone who presumably has the parenting resources or parenting desire the pregnant person lacks. Alternatively, some who think abortion is unethical reach the same conclusion based on what is called “potential theory”: the fact that an embryo will later achieve the same moral status as you and me means we must treat it in accord with its future (potential) status.

“Potential child” has been endlessly debated as a morally relevant category. However, our long tradition of imagining women as mindless vessels has led us to undertheorize “potential mother” as a morally relevant category, separate from an assumption that it leads directly to an antiabortion position. Grounding analysis in an experiential approach allows us to name something rarely articulated and embarrassingly obvious: that becoming a mother through pregnancy and birth is a gradual process with moral implications, just as developing from blastocyst to baby is a gradual process with moral implications.

Therefore, the new possibility I'm raising here is that the moral seriousness of motherhood might begin at conception because an embryo has the potential to become a person, but not because this potentiality vests full moral status in an embryo. Instead, it's because the embryo's potentiality requires the pregnant person to begin making decisions and taking actions that will shape the future. In fact, the belief that an embryo is *not* the moral equivalent of a person, combined with the knowledge that it *will* become a baby, child, and adult if its development is not interrupted, is exactly what motivates abortion decisions. Similarly, every pregnant woman who avoids alcohol, blue cheese, and bungee jumping for the benefit of their developing embryo or fetus or who gets a crib and car seat for the benefit of the baby it will become is responding as a mother before they fit the dictionary definition of one. In this liminal state of “anticipatory motherhood,” I argue, the pregnant person has both obligations and options.^19^

I object to outsiders describing pregnant women seeking abortion as “mothers” in an attempt to convert their fetuses into “children.” (For example, in a dissent in *Roe v. Wade*, Justice Byron White calls the person seeking abortion “the mother” or “the pregnant mother,” whereas the majority calls her “the pregnant woman.”^20^) However, my thinking about how pregnant women seeking abortion might conceptualize themselves was enriched when I read 394 narratives written by abortion patients before or after their procedure in notebooks that one clinic set out for this purpose.^21^ In 2022, my coauthor and I reported that approximately one‐quarter of the writers framed themselves as being in a mother‐child relationship with the embryo or fetus they were aborting that day. In an overlapping category, half of these writers expressed what we called “maternal reasoning,” saying that their existing children or their potential children (either the embryo or fetus they carried or children they hoped to have in the future) would be better off as a result of their abortion. These narratives move us from politics to experience and document that women's consideration of whether abortion is in the best interest of their embryo or fetus goes beyond patients with medical reasons for abortion. The narratives suggest that some people ending pregnancies simultaneously inhabit the identities of abortion patient and mother or future mother, that these overlapping identities are important to them, and that many abortion patients understand their decision to end a pregnancy as part of family formation and preservation. To me, this is further evidence that the moral seriousness of motherhood begins at conception regardless of whether the pregnant person decides to abort or deliver.

Another crucial and rarely recognized fact is that some women believe that abortion is a morally permissible way to avoid parenthood but that adoption is not, again illustrating how the moral seriousness of motherhood can be seen as beginning at conception even if embryos and fetuses are not regarded as having the full moral status of a person. The belief that adoption is an immoral choice emerges in two academic studies—one of pregnant women who had abortions^22^ and a second of pregnant women who carried to term.^23^ In the first study, thirty‐eight women who had an abortion were asked about their reasons in 2004. Motherhood as a reason for abortion was not in the interview guide, but thirty‐six of the thirty‐eight subjects spontaneously raised it, and that led to a secondary analysis of those answers.^24^ They also were not specifically asked about adoption, but over one‐third volunteered that “they had considered adoption and concluded it was a morally unconscionable option because giving one's child away is wrong.”^25^ The second study, conducted from 2008 to 2016 and known as the Turnaway Study, followed 161 women who delivered babies after they were denied an abortion because they were past a clinic's gestational limit. This seems like the population most likely to turn to adoption, but surprisingly,^26^ only 9 percent chose to relinquish their “unwanted” children.^27^ Why did 91 percent choose parenting? Some said it was because they believed that placing their baby for adoption was an abdication of responsibility and they would feel guilty if they did it. (For others, the reason they didn't pursue adoption was the bond they felt after the birth: “[a]fter meeting their children, adoption became unimaginable.”)^28^ Interestingly, a study conducted by the conservative Family Research Council supports these findings. This 2000 report on why pregnant women in crisis pregnancy centers refused to (or were reluctant to) consider adoption found that they equated adoption with abandonment and deception and considered it an unbearable sacrifice.^29^

I am not arguing that this view of adoption is correct or incorrect. I am arguing that, in a pluralistic society, these women's beliefs are entitled to respect. The women are asserting the moral seriousness of motherhood, and their view suggests that involuntary adoption is a harm akin to involuntary abortion. The moral seriousness of motherhood begins at conception in anticipation of the altered moral status that will come later for the embryo that will have the rights of a baby if that pregnancy is continued to delivery. But the pregnant woman changes at birth too—and, critically, this is true even if she doesn't raise her baby, according to Maureen and almost all the women Fessler and Sisson interviewed. Grounding analysis in an experiential approach also suggests that, even when the activity of mothering ends at birth, the identity of being a mother often continues. This splitting of identity and role helps explain why involuntary adoption can be such a painful violation for these birth mothers. As one of Sisson's subjects put it, “Don't look at [adoption] like you will be able to go back to your life because you won't be able to. You will now be a mother without a child.”^30^ Or as Fessler reports, “Women who said they never entertained the idea of parenting at the time of their surrender often described the same lifelong grief as those who fought to bring their baby home.”^31^ Maureen says the loneliness made her pregnancy difficult, but so did the love. “It's very hard to carry a pregnancy and have no feelings for this baby that's growing inside of you,” Maureen explained. “And in my teenage experience, I kind of fell in love with this baby. I talked to her; I named her.” Maureen said that some of her pro‐choice friends worry that describing that love grants fetuses a kind of personhood, but as an obstetrician who performs abortions, Maureen doesn't see it that way. “You can have feelings for this fetus that's growing and at the same time know you're not going to keep that fetus.”^32^ Maureen did not want to become a mother at nineteen. Yet a maternal bond she did not want to create developed, and then was disrupted—she fell in love with the baby she delivered, hoped her daughter would return to her someday, and eagerly embraced Deanna when she did.

## Case 2: A Pre‐***Roe*** Adoptee

Chicago real estate agent Caroline Moellering was born and adopted in Fort Wayne, Indiana, in 1965. As a little girl, her resemblance to Samantha Stevens on *Bewitched* convinced her that Samantha was her birth mom, and Caroline worked on making candlesticks and vases levitate for confirmation. In 2019, at age fifty‐four, after both her parents had passed away, she did the twenty‐first‐century version of Deanna's call to the adoption agency: she sent a DNA sample to 23 and Me and used Ancestry.com. Within a few months, she had located her birth mother. Caroline sent her a letter with photos of herself and her children. Yet, unlike Maureen, not every pre‐*Roe* birth mother wants to be found. Caroline was disappointed by the reply, which she characterizes as dismissive and unkind. “When the adoption took place I was assured that the information provided would remain secured and my identity anonymous,” Caroline's birth mother wrote. “That promise no longer holds true it seems, now that ancestry.com and others have emerged…. I will continue to keep you in my thoughts and wish you the very best. I trust that in return you will respect my privacy and honor my request for NO further communication.” Reading *The Girls Who Went Away* helped Caroline feel “somewhat forgiving” about this reply.

Caroline got a different response from her biological father, and she felt like his open arms were partly a function of gender. “He was like, ‘Oh boy! What took you so long? We've been waiting.’ But he didn't have really any skin in the game. He got to run around with his normal body, no belly showing, and go back to the fraternity house, while this poor woman had to be carted up to Indiana [to a family friend's home]. There's much more displacement when you're the woman.”

## Resisting the Hijacking‐of‐Adoptees’‐Lives Antiabortion Argument

Like birth mothers, adoptees are, I think, an interesting and underexamined group in the abortion debate.^33^ Adoptees like Caroline and Maureen's birth daughter, Deanna, seem like poster children for the antiabortion bumper stickers that ask, “Aren't you glad your mom didn't have an abortion?,” but that's not their perspective. Caroline is strongly pro‐choice and thinks it is senseless to ponder what would have happened if abortion had been legal when she was conceived “because if I wasn't here, I wouldn't be here. No one would miss me,” Caroline said. “I'm here. But I think I'm here because of coercion, societal coercion. I also feel like nine months of my pre‐life existence caused a lot of upset for the woman who carried me, and I think it would have been nice if she could have had her life without that emotional turmoil.” Deanna also rejects what I think of as “the grateful fetus narrative.” “I just wouldn't have existed,” Deanna stated. “Even as a fetus, I'm not really a person, or I wouldn't have been really a person. The person is when you interact and love each other and know each other,” she said. When *Roe v. Wade* was reversed, even Shelley Lynn Thornton, the daughter of Ms. Roe (Norma McCorvey), added her name to the chorus of pro‐choice adoptees.^34^ And an adoptee profiled in a *New Yorker* article on adoption reported that she found it tricky to be an antiabortion Christian among adoptees because “[i]t was striking how pro‐choice the adoptee community was.”^35^ Caroline is disgusted by the rhetorical “Aren't you glad?” question. “The person who puts that sticker on his or her car is not thanking his or her birth mother. That person is shaming those who have made other, until recently, completely legal choices.”

Lisa Wool‐Rim Sjöblom is an artist and transnational Korean adoptee^36^ who takes “great offence when lobbyists hijack my life as an adoptee and think it appropriate to steal my voice to support their abusive agendas.”^37^ She responded to the *Dobbs* ruling with a comic titled “Would you have rather been aborted?” and in an interview explained, “Quite often, it isn't so much of an honest question but more a way of gaslighting adoptees who are voicing their critique. It's a way of telling you that you're more or less ‘walking dead’ and therefore should stay quiet and be grateful.”^38^ She says her answer to the question would be to “have given her [mother] options, control, and self‐determination…. [If] she had wanted an abortion, she should have had the right to one. The person—me—who never was would never have known and could therefore not be here to answer that question.” Wool‐Rim Sjöblom adds that many birth mothers “were forced to carry to term, knowing that their children would be taken from them straight after birth…. This is what we should be talking about, not whether adoptees would rather have been aborted.”^39^

Those who oppose legal abortion seem to claim special knowledge of what embryos and fetuses with no conscious desires want, and they claim to speak on their behalf. Yet we are all former fetuses with equal knowledge and equal standing to speak. Some of us share the views of Deanna, Caroline, and Wool‐Rim Sjöblom; others think embryos or fetuses have a high moral status but think the moral status of the person in whom they live is even higher; others think embryos or fetuses have a moral status that is so high (or that the moral status of adult women is so low) that ending pregnancy is almost always impermissible. This range of beliefs perfectly fits the pluralism and freedom of conscience that the U.S. Constitution has traditionally protected and that was embraced by the late twentieth‐ and early twenty‐first‐century legislative trend of repealing morality laws. When the stakes are high and reasonable people disagree, the government stays out of our personal lives. The Supreme Court reversed this trend in *Dobbs*, inviting state governments back into the business of passing morality laws that decimate private decision‐making around family formation.^40^

It is amazing that the number of U.S. abortions did not go down in the first year after *Dobbs* given the number of states that introduced abortion bans.^41^ The phenomenal effort many pregnant women in ban states are making to travel to standard‐of‐care states or to obtain medication abortion by mail may be a new signifier of the moral seriousness of motherhood; those who do not want the role now, ever, or again have forcefully resisted their state's efforts to force it on them. Could the determination of abortion patients, combined with the dedication of reproductive health providers and advocates, mean a wave of involuntary adoption is not on the horizon?

Perhaps, but those high numbers might not last. The number of U.S. abortions may go down as more states enact bans and travel distances increase, or if donations to the private charitable funds that finance travel continue to decrease as our collective attention drifts to other causes, or if the federal government enforces the Comstock Act's prohibition on mailing anything related to abortion (including pills)—or any number of additional scenarios.

An additional question embedded in the before‐and‐after comparison is the “right” number of abortions. The U.S. abortion rate would likely be higher if we had unfettered access. As mentioned earlier, before *Dobbs*, due to a lack of Medicaid coverage, women in Louisiana brought to term approximately three thousand unwanted pregnancies per year, and in each of the other thirty‐two states with the same Medicaid policy, a comparable proportion of women likely had the same experience. We do not know these mothers’ relinquishment rate, but the adoptions that might exist in this group are also a result of laws preventing abortion access. Similarly, the Turnaway Study mentioned earlier followed one group of women who delivered because they were too far along for an abortion at the clinic they went to and they couldn't make it to another, and that group's 9 percent adoption rate (which is the same as the pre‐*Roe* rate) is driven by laws preventing abortion access later in pregnancy. As a result, it is probably inaccurate to claim that *Roe* stopped involuntary adoption driven by legislative coercion. In reality, *Roe* only decreased it.

Whatever the number, there's no going backward to right the wrongs of involuntary adoption. Janet Mason Ellerby wrote that, when she was reunited with her daughter thirty‐five years after her relinquishment, she was certain she would be healed and transformed, but that's not how it ended for her or for other birth mothers she knew in the same situation. “We were unprepared when our longing resumed. We were frustrated when our regret persisted. We were shocked when our sorrow returned. Our happy ending dissolved. We still longed for the past. The deed could not be reversed. The baby could not be restored. The longing could not be assuaged. The adult child may have lovingly returned, but the first loss and the deep‐seated sorrow would not be mollified.” For Ellerby, “resentment and regret [are] sentiments that have not substantially subsided in over fifty years.”^42^

Maureen wanted an unattainable abortion rather than the unattainable parenting Ellerby sought. Yet tellingly, Maureen has comparable feelings about her lost years of contact.

## Twenty Years Gone

In the thirty‐three years since Maureen and Dillon met Deanna, they have gotten together every year or two.

“You and Dillon are tight,” Deanna said to her birth mother. “I didn't feel left out, but I didn't have that same depth of connection.”

“I feel like my intimacy is different with each of you,” Maureen responded. “Because I raised Dillon, we grew up together, Dillon knows me. And it's nature nurture.” Maureen turned to Deanna. “But I feel like I have this nature thing with Deanna. From the minute I met you, you were so much like me, and in some ways, I think we're more alike than I am with Dillon. Like our ideas about parenting—eh, just give 'em the keys—or our sense of humor,” Maureen said. “It always kind of amazes me that, not having lived your first twenty years together, that we're as alike as we are, and the only explanation I have is genetics. I mean, her whole sense of energy. I'm like, she may be a Venzke, but there's a Paul in there!” The women laughed.

“But I do want to say, whenever I look at Deanna, I miss that twenty years,” Maureen said. “I can't have that twenty years, but I miss the twenty years.” For the first time in our afternoon together, Maureen began crying. Both daughters slowly rose for tissues, eyes locked on the woman who conceived, gestated, and birthed them. “Adoption can be a great thing; adoption can be a traumatic thing,” Maureen said, dabbing her tears. “You just can't legislate it.”

## Acknowledgments

Thanks to M. K. Czerwiec, Megan Crowley‐Matoka, and Debjani Mukherjee for helpful comments on an earlier draft.
